# Assessment of Long-Term Badminton Experience on Foot Posture Index and Plantar Pressure Distribution

**DOI:** 10.1155/2019/8082967

**Published:** 2019-01-02

**Authors:** Ping Huang, Minjun Liang, Feng Ren

**Affiliations:** ^1^Faculty of Sports Science, Ningbo University, Ningbo, China; ^2^Research Academy of Grand Health, Ningbo University, Ningbo, China

## Abstract

This study was aimed to analyze the foot posture index and plantar pressure characteristics of fifteen badminton players and fifteen controls. The hypothesis was that people with the habit of playing badminton would be significantly different with nonplaying people in foot posture index, 3D foot surface data, and plantar pressure distribution. Nine regions of plantar pressure were measured by using the EMED force platform, and badminton players showed significantly higher peak pressure in the hallux (*p* = 0.003), medial heel (*p* = 0.016), and lateral heel (*p* = 0.021) and force-time integral in the hallux (*p* = 0.002), medial heel (*p* = 0.026), and lateral heel (*p* = 0.015). There is no asymmetrical plantar pressure distribution between the left foot and the right foot of players. The mean foot posture index values of male and female badminton players are 5.2 ± 1.95 and 5.7 ± 1.15, respectively, and comparatively, those values of male and female controls are 1.5 ± 1.73 and 1.7 ± 4.16, respectively. This study shows that significant differences in morphology between people with the habit of playing badminton and people without that habit could be taken as a factor for a future study in locomotion biomechanics characteristics and foot shape of badminton players and in a footwear design in order to reduce injury risks.

## 1. Introduction

Badminton attracted extensive participation when it was introduced to the Barcelona Summer Games in 1992 [1]. With the prevalence of badminton, the International Badminton Federation reported that there were about 200 million people playing badminton around the world [2]. Due to easy learning rules, low-cost equipment, and small playing court, badminton appeals participants of different ages, economic conditions, and physical capabilities [3]. Long-time badminton sport will cause a series of biological adaption and modifications to the motor system.

Long-time badminton sport has adaptive influence on the foot. Biological adaptation is a feature of phenotypic characteristics of organisms adapting to the selection requirement of the environment. According to the features of sports, biological adaptive modifications can be short term or long term, which indicates adaptive modifications on all body levels require complicated efforts. High-level sports performance is based on the biological adaptation degree of the body. The continuous improvement of sport level is the basis of physiological support [4]. Previous studies showed that different foot shapes have different foot functions. Although presenting the same anatomical features, human foot has various shapes and biomechanical characteristics [5–7]. Players of different races and different sports levels have different plantar pressures, foot shapes, and foot functions [8]. Previous studies have shown that by understanding the characteristics of foot pressure distribution, it is possible to effectively optimize technical movements, reduce foot injuries, and improve the design of special shoes [9]. A report indicated that when professional badminton players finish competitions, stress gathers in their Achilles tendon and anterior knee tendons, especially in the dominant lunge leg [10]. High plantar pressure serves as an implicit causation of sports injuries to lower limbs [11]. As a result, recognizing the impact forces and features of plantar pressure distribution would contribute to finding the preseasons of sports injuries.

Different sports have different technical posture features, which may result in certain foot shape comparing to other foot shapes. The foot posture index (FPI), as an effective measurement for the quantization of standing foot posture, is relatively simple and fast to determine foot posture [12, 13]. FPI values of special postures in different sports are different [14]. Previous studies showed that FPI values of a runner, basketball player, and handball player are significantly different, mainly caused by talar head position and talonavicular [15]. The FPI index of basketball players in different positions is related to lower limb injuries. After running for a long time, foot posture and plantar pressure overall decrease in peak and mean plantar pressure was revealed. The FPI value will be different during sport with higher intensity [16]. In the field of badminton study, there are few studies about FPI and foot shape. Sports injuries may be reduced when designers design shoes according to different foot shapes [17, 18].

However, badminton players are rarely taken as the study participant. This study recruited 15 badminton players and 15 normal people as the control. In the meantime, data of kinetics and foot shape of the 30 participants are collected, aiming to find the characteristics of kinetics and foot shape. The hypothesis is that badminton players will be significantly different from controls in plantar pressure-based foot morphology and posture characteristics.

## 2. Methods

### 2.1. Participants

A total of 30 participants, including 15 badminton players and 15 controls, participated in the experiment approved by the local ethics committee. The participants signed a consent form and were told about the requirements and procedures before the experiment. Badminton players have several years of badminton exercising or playing habits and play more than one hour every time. Controls do not have badminton exercise habits. In the past half year, participants do not have any injuries in both upper and lower limbs. Their basic demographics are shown in Table 1.

### 2.2. Design and Procedures

#### 2.2.1. Plantar Pressure Measurements

**Figure 1:**
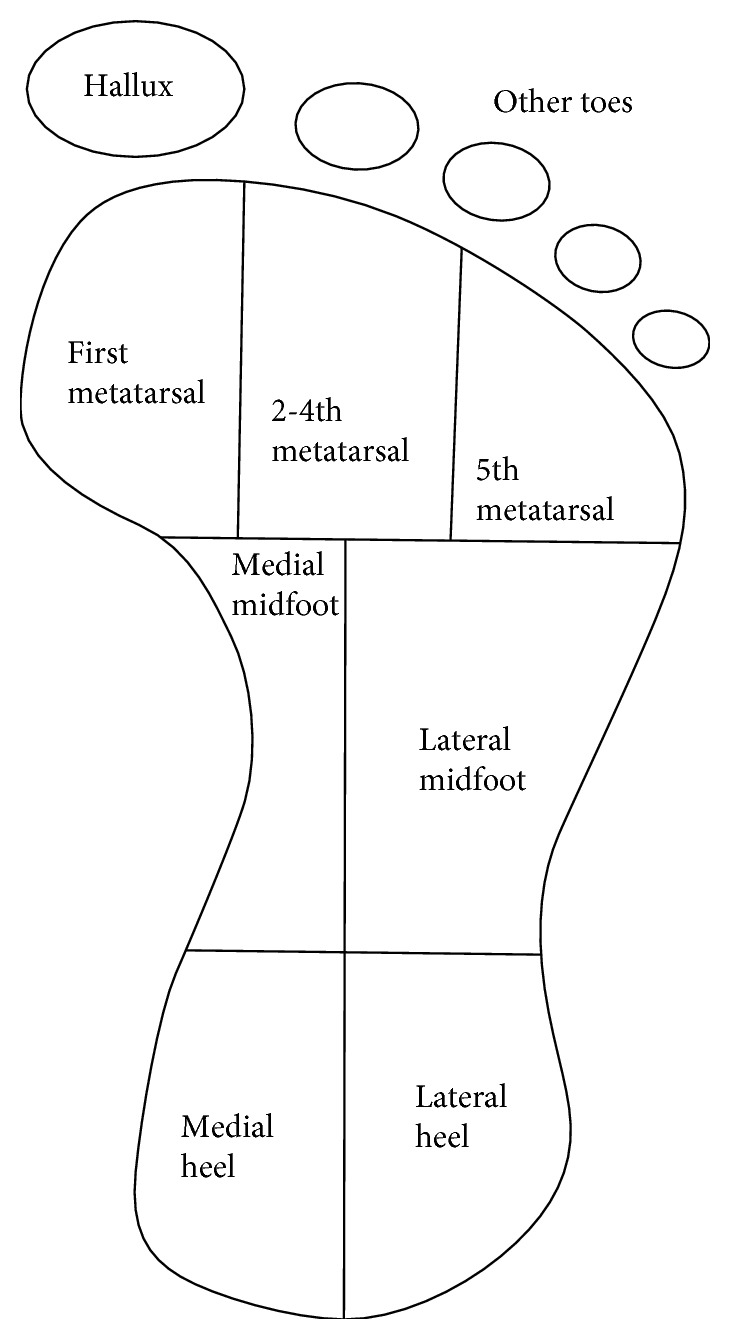
The foot segments (nine in total) used by the EMED pressure platform.

An EMED pressure platform was used to record plantar pressure at 50 Hz (Novel, Germany). The platform was placed on the ground at the center of an 8-meter walkway. The participants were trained to walk and run on the platform before the test, and then every participant was required to walk and run on the platform five times. Every participant started walking and running approximately five steps before contacting the platform and contacted the platform at the sixth steps, then continued to walk and run. All tests were supervised, using a timer to test the time during a certain distance and calculate the average speed of every subject in each trial, and then the date will not be used if the average speed in the trial deviates over ±5% from the certain walking and running speeds. Participants would be asked to do the task one more time. The plantar pressure of every participant was recorded more than five times, and the averaged value was used for analysis. After data collection, peak pressure, contact area, and pressure time integral were obtained from the plantar pressure measurement system. The footprint was divided into nine anatomical segments (Figure 1): hallux (H), other toes (OT), first metatarsal (M1), second and fourth metatarsals (M24), fifth metatarsal (M5), medial midfoot (MM), lateral midfoot (LM), medial heel (MH), and lateral heel (LH).

### 2.3. Foot Posture Index

**Figure 2:**
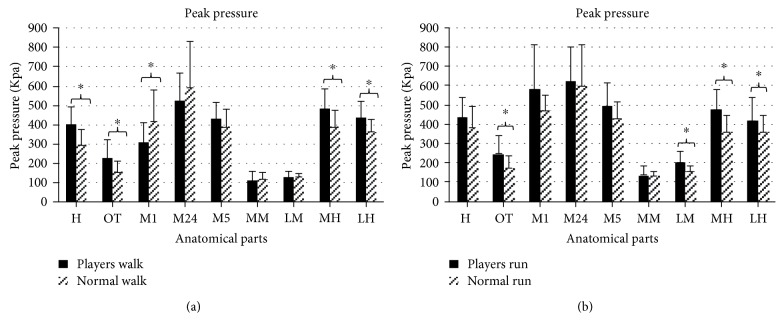
The peak pressure in nine anatomical parts with an illustration of existing significance (∗ indicates *p* < 0.05).

Foot posture index (FPI), as a clinical tool, can quantify the angle a foot can be pronated and supinated to [13, 19]. This is a relatively easy, fast, and reliable method [20]. The FPI was assessed in standing using the original protocol with the six items [12]: (1) talar head palpation, (2) curvature at the lateral malleoli, (3) inversion/eversion of the calcaneus, (4) talonavicular bulging, (5) congruence of the medical longitudinal arch, and (6) abduction/adduction of the forefoot on the rearfoot (Figure 2). Each item was scored on a scale of −2, −1, 0, +1, and +2 (0 for neutral, −2 for clear signs of supination, and +2 for clear signs of pronation), and all scores were summed. The final score ranged from −12 to +12; a larger positive value means a more pronated foot. There are no significant differences in the FPI between the right foot and the left foot in asymptomatic individuals [21]. The FPI values and the plantar pressures only used the right foot measurements to avoid breaching assumptions of statistical independence in bilateral limb studies [22]. The FPI was evaluated by an experienced professional who did not know the purposes of the study and the participant identity and only sees the foot and 10 cm of the shank [23].

The BMI (body mass index) means the body weight (kg) divided by the squared body height (m^2^). The World Health Organization (WHO) regards BMI values between 18.5 and 23.9 as normal, values below 18.5 as underweight, and values over 30 as obese. See Table 1. As all participants' BMI were in the normal range, the foot shape changes due to different body weights or load-bearing conditions and different stature can be negligible bearing their own body weight [24, 25].

### 2.4. Statistical Analyses

The normality of variables in this experiment was checked before statistical analysis. An independent sampled *t*-test was used for the peak pressure, contact area pressure time integral, and FPI data analysis. The effect size was calculated according to Cohen's *d* used for comparing the differences in the mean value of the two groups. The statistical power of the analysis was calculated using NCSS-PASS 16.0 software (Table 2). We established new variables on the basis of the foot morphological values measured and did not consider participants' weight. We created new variables of length, width, ball, waist girth, and short heel to compare the athlete foot with the normal foot morphological characteristics. Shortly, the new variables were obtained using the formulae as follows:

1. Ratio: length/width
2. Ratio: ball/waist girth
3. Ratio: short heel/length
4. Ratio: short heel/width

All statistical analyses were carried out by using SPSS 17.0 (SPSS Inc., Chicago, IL, USA) with significance level settings at *p* < 0.05.

## 3. Results

**Figure 3:**
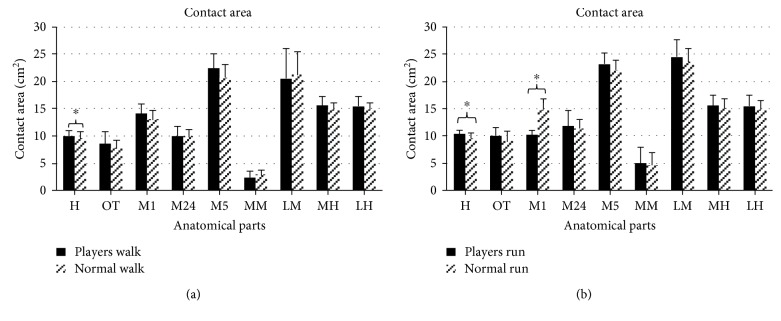
The contact area in nine anatomical parts with an illustration of significance (∗ indicates *p* < 0.05).

**Figure 4:**
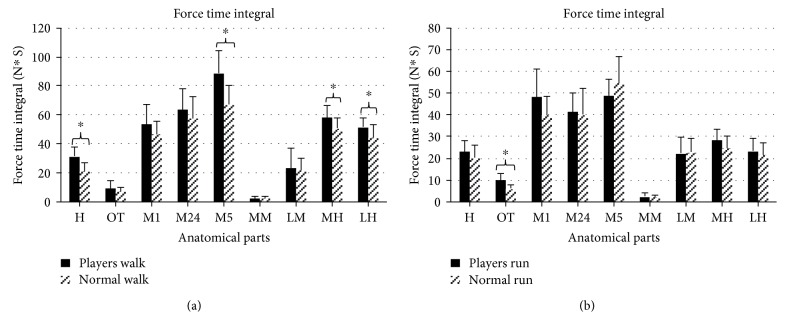
The force-time integral in nine anatomical parts with an illustration of significance (∗ indicates *p* < 0.05).

For the plantar pressure, the mean and standard deviation (SD) values of foot loading distribution characteristics are shown in Figure 2 (peak pressure), Figure 3 (contact area), and Figure 4 (force-time integral) for every anatomical part. The acronyms MH, LH, MM, LM, M1, M24, M5, H, and OT stand for the medial heel, lateral heel, medial midfoot, lateral midfoot, first metatarsal head, second and fourth metatarsal heads, fifth metatarsal head, hallux, and other toes of badminton players and normal people.

Figure 2 displays the mean (SD) values of peak pressure in badminton players and in those without the habit. During walking tests, the significance values (*p*) of nine anatomical parts are 0.003^∗^ (H), 0.021^∗^ (OT), 0.047^∗^ (M1), 0.394 (M24), 0.217 (M5), 0.375 (MM), 0.887 (LM), 0.016^∗^ (MH), and 0.021^∗^ (LH) with higher variance exhibited in H, OT, M1, MH, and LH. During running tests, the significance values (*p*) of nine anatomical parts are 0.136 (H), 0.014^∗^ (OT), 0.104 (M1), 0.932 (M24), 0.132 (M5), 0.963 (MM), 0.047^∗^ (LM), 0.006^∗^ (MH), and 0.036^∗^(LH). Peak pressures in the forefoot and rearfoot of badminton players are significantly larger than those of people without that habit. In Figure 3, the contact areas are depicted. When the participants are walking, the significance values (*p*) from independent sampled *t*-tests are 0.034^∗^ (H), 0.392 (OT), 0.664 (M1), 0.976 (M24), 0.133 (M5), 0.502 (MM), 0.983 (LM), 0.318 (MH), and 0.138 (LH). Professional and amateur players show significant differences only in the hallux during the walking test, and the differences in other toes are not obvious. When the participants are running, the significance values (*p*) are 0.042^∗^ (H), 0.076 (OT), 0.000^∗^ (M1), 0.773 (M24), 0.114 (M5), 0.940 (MM), 0.443 (LM), 0.114 (MH), and 0.074 (LH). Significant differences in the contact area between professional players and amateur players appear in the inside of the forefoot during the walking test. In Figure 4, the force-time integrals (impulse) are illustrated. When the participants are walking, the significance value (*p*) are 0.002^∗^ (H), 0.095 (OT), 0.138 (M1), 0.178 (M24), 0.002^∗^ (M5), 0.562 (MM), 0.683 (LM), 0.026^∗^ (MH), and 0.015^∗^ (LH). The force-time integrals to H, M5, MH, and LH of badminton players are significantly larger than those of controls. When the participants are running, the significance values (*p*) are 0.061 (H), 0.001^∗^ (OT), 0.085 (M1), 0.650 (M24), 0.279 (M5), 0.653 (MM), 0.518 (LM), 0.079 (MH), and 0.299 (LH).

30 participants include 15 people with the habit of playing badminton and 15 people without the habit of playing badminton. The mean FPI values of males and females with the habit of playing badminton are 5.2 ± 1.95 and 5.7 ± 1.15, respectively, and that of males and females without the habit of playing badminton are 1.5 ± 1.73 and 1.7 ± 4.16, respectively. No mean differences in the FPI were shown between the right foot and the left foot. The mean FPI of the study group are displayed in Table 3.

## 4. Discussion

The purpose of this study was to identify that biological adaptation in people without the habit of playing badminton will be significantly different from people having the habit of playing badminton in plantar pressure and kinematics. The experimental results support this hypothesis. The characteristics of plantar pressure distribution and that of foot shape are significantly different between people with many years of badminton playing habit and those without that habit when they walk and run. These parameters should be considered in future sports intervention and sports equipment design.

Long-time sport can improve physical activity level, and supercompensation theory and adaptation theory have explained the changes of people's sports ability when training [26]. Reports said that research on the plantar pressure of athletes may optimize technology, promote footwear design, and reduce the risk of foot injuries [27]. Therefore, the data of plantar pressure in walking and running are collected and analyzed. In terms of plantar pressure distribution, peak pressures of H, OT, M1, MH, and LH of badminton players are significantly higher than controls in walking. These features are related to the habit of landing on the balls of their feet when people play badminton. The contact area and force-time integral of H show a marked difference, demonstrating that the metatarsal head and lateral heel are areas with the highest pressure; thus, different areas of outsoles need different materials for dispersing pressure [28].

In actual badminton sports movement, the in-shoe peak plantar pressures in left- and right-forward lunges were investigated [29]. Thus, differences in the plantar pressures among lunges of different directions may be latent risks for badminton players to sustain injuries to their lower extremities [30]. This research probed into plantar pressure distribution of different areas of both the right foot and the left foot of badminton players and controls when they are walking and running. The analysis of plantar pressure of the right and left feet shows that there is no significant difference between them, which is in accordance with the analysis of muscle force around the ankle; that is, the difference in the bilateral antagonist muscle ratio is caused by the difference in force using the method instead of that in bilateral muscle force [31].

Results show the correlation between FPI and plantar pressure [32]. Many researches have estimated the pressure distribution of flatfeet, and some of them aimed at identifying the normal value [33]. Therefore, the FPI is a more intuitive and reliable index for selecting athletes. Results of our research show that the mean FPI values of males and females with the habit of playing badminton are 5.2 ± 1.95 and 5.7 ± 1.15, respectively, and that of males and females without the habit of playing badminton are 1.5 ± 1.73 and 1.7 ± 4.16, respectively.

Fatigue caused by sports will lead to changes in the plantar pressure. Changes in foot posture after running were studied and combined with the plantar pressure model, indicating that heel strike posture during running is related to plantar pressure distribution. The investigation can help understand foot function, prevent sport-related injury, and design effective foot type orthodontic appliance [34, 35]. This method was rarely used in badminton research before. In the future, changes in the foot posture index after playing badminton can be taken as a factor to study the biological adaptation of foot function of badminton players after long-term badminton sports.

The foot shape index is used to evaluate foot function and footwear design. In general, length, width, and height are regarded as the standards to test whether the footwear is fit to the foot [36, 37]. However, owing to that foot height is not directly related to foot length, the method of grading shoes by increasing height or scaling according to foot length is undesirable [24, 25]. Common indexes of the footwear design are foot length, heel width, forefoot width, and midfoot width. The research aimed at providing reference indexes for a badminton footwear design by setting specific ratios according to foot shape features. Results show that there is no significant difference in foot shape between people with many years of badminton playing habit and those without that habit, but data of this research, combined with data of plantar pressure distribution and FPI, can be taken as reference indexes for footwear design. Further research can use the arch index as the reference index. Findings show that the arch index and plantar pressure characteristics of badminton players were usually categorized as high-arched supinator [38]. In a future study, the changes of FPI values when performing special movements to certain intensity in a badminton sport can be used to analyze the cause of sports injuries.

In conclusion, this research studied that people having the habit of playing badminton will be significantly different from people without the habit of playing badminton in plantar pressure and kinematics. According to the difference in plantar pressure distribution and foot shape feature through this research, peak pressures of H, OT, M1, MH, and LH of badminton players are significantly higher than controls in walking and running. The combination of FPI and foot shape index may provide implications for the instruction of amateur players, designing sports equipment, improving sports technologies, and reducing sport-related injuries.

## Figures and Tables

**Table 1:** The basic demographics of habitual badminton players and normal people.

	Badminton players		Normal players	
	Male	Female	Male	Female
Age (years)	22 ± 2.8	21 ± 1.0	24 ± 1.2	23 ± 1.0
Weight (kg)	69.8 ± 6.5	51.7 ± 2.9	67 ± 6.1	61 ± 12.1
Height (m)	175 ± 4.5	162 ± 2.9	173 ± 4.1	163 ± 6.4
BMI (kg/m^2^)	21.66 ± 1.38	19.75 ± 0.39	21.99 ± 1.46	20.58 ± 1.29
Badminton experience (years)	5.5 ± 2.8	6 ± 0	0	0

Note: mean ± standard deviation; BMI—body mass index.

**Table 2:** G power for the foot pressure.

	Peak pressure				Contact area				Force-time integral			
	Walk		Run		Walk		Run		Walk		Run	
	Effect size	Power	Effect size	Power	Effect size	Power	Effect size	Power	Effect size	Power	Effect size	Power
H	0.52	0.84	0.23	0.8	0.21	0.8	0.31	0.8	0.64	0.88	0.29	0.81
OT	0.4	0.82	0.42	0.81	0.19	0.8	0.28	0.81	0.28	0.8	0.61	0.85
M1	0.37	0.82	0.31	0.81	0.18	0.8	0.83	0.93	0.31	0.81	0.37	0.82
M24	0.18	0.8	0.07	0.8	0.01	0.8	0.08	0.8	0.21	0.8	0.07	0.8
M5	0.22	0.81	0.3	0.81	0.32	0.81	0.28	0.8	0.6	0.83	0.26	0.81
MM	0.12	0.8	0.02	0.8	0.2	0.8	0.08	0.8	0.07	0.8	0.1	0.8
LM	0.09	0.8	0.48	0.83	0.06	0.8	0.16	0.8	0.09	0.8	0.03	0.8
MH	0.44	0.81	0.53	0.8	0.26	0.8	0.13	0.8	0.42	0.82	0.34	0.8
LH	0.44	0.81	0.29	0.8	0.24	0.8	0.18	0.8	0.41	0.81	0.16	0.8

**Table 3:** Mean values for the foot posture index.

	Habitual players		Normal players	
	Male	Female	Male	Female
FPI	5.2 ± 1.95	5.7 ± 1.15	1.5 ± 1.73	1.7 ± 4.16
L/W ratio	2.3 ± 0.26	2.2 ± 0.22	2.2 ± 0.32	2.1 ± 0.10
B/W G ratio	1.0 ± 0.09	1.0 ± 0.02	1.0 ± 0.02	1.0 ± 0.01
S H/L ratio	1.3 ± 0.04	1.3 ± 0.06	1.3 ± 0.04	1.3 ± 0.04
S H/W ratio	2.9 ± 0.30	2.8 ± 0.26	2.7 ± 0.37	2.6 ± 0.13

Note: values are presented as the mean ± standard deviation. L/W ratio, length/width; B/W G ratio, ball/waist girth; S H/L ratio, short hell/length; S H/W ratio, short hell/width.

## Data Availability

The data that support the findings of this study are available from the corresponding author upon reasonable request.
